# Comparison of five pretreatments for the production of fermentable sugars obtained from Pinus pseudostrobus L. wood

**DOI:** 10.17179/excli2014-613

**Published:** 2015-03-13

**Authors:** Juan Carlos Farías-Sánchez, Javier López-Miranda, Agustín Jaime Castro-Montoya, Jaime Saucedo-Luna, Artemio Carrillo-Parra, Pablo López-Albarrán, María Guadalupe Pineda-Pimentel, José Guadalupe Rutiaga-Quiñones

**Affiliations:** 1 Facultad de Ingeniería en Tecnología de la Madera Edificio D y V1, CU, Universitaria Universidad Michoacana de San Nicolás de Hidalgo, Av. Fco. J. Múgica S/N. Col. Felicitas de Río, Morelia, Michoacán, C.P. 58040, México; 2 Departamento de Ingenierías Química y Bioquímica, Instituto Tecnológico de Durango, Blvd. Felipe Pescador 1830 Ote., Col. Nueva Vizcaya, Durango, Durango., C.P. 34080, México; 3 Facultad de Ingeniería Química, Edificio D y V1, CU, Universitaria Universidad Michoacana de San Nicolás de Hidalgo, Av. Fco. J. Múgica S/N. Col. Felicitas de Río, Morelia, Michoacán, C.P. 58040, México; 4 Facultad de Ciencias Forestales, Universidad Autónoma de Nuevo León, Carretera Nacional núm. 85, km 145, Linares, Nuevo León, C.P. 67700, México

**Keywords:** reducing sugars, enzymatic hydrolysis, acid hydrolysis, pretreatments, pine sawdust, Pinus pseudostrobus

## Abstract

To benefit from the use of a waste product such as pine sawdust from a sawmill in Michoacán, Mexico, five different pretreatments for the production of reducing sugars by enzymatic hydrolysis were evaluated (sodium hydroxide, sulfuric acid, steam explosion, organosolv and combined method nitric acid / sodium hydroxide). The main finding of the study was that the pretreatment with 6 % HNO_3_ and 1 % NaOH led to better yields than those obtained with sodium hydroxide, dilute sulfuric acid, steam explosion, and organosolv pretreatments. Also, HNO_3_ yields were maximized by the factorial method. With those results the maxima concentration of reducing sugar found was 97.83 ± 1.59, obtained after pretreatment with 7.5 % HNO_3_ at 120 °C for 30 minutes; followed by 1 % of NaOH at 90 °C for 30 minutes at pH 4.5 for 168 hours with a load enzyme of 25 FPU/g of total carbohydrates. Comparing the results obtained by the authors with those reported in the literature, the combined method was found to be suitable for use in the exploitation of sawdust.

## Introduction

Ethanol can be produced by fermentation of sugars presents in vegetables products (cereals, beet, cane, sorghum, and other biomasses); these sugars are present in the forms of saccharose, starch, hemicelluloses, and cellulose. The product of this fermentation process is hydrated alcohol containing approximately 5 % of moisture. After a dehydration step, the alcohol can serve as a vehicle fuel (SEMARNAP, 2000[27]). 

Forest lands, and particularly timberlands, have the potential to sustainably produce close to 370 million dry tons of biomass annually. This estimate includes the residues generated in the manufacture of various forest products and the residues generated in the use of manufactured forest products. It also includes the harvest of wood for various residential and commercial space-heating applications. With the exception of urban wood residues, most of these sources of forest biomass are currently being utilized and there are significant efforts under way to use these resources much more efficiently (Perlack et al., 2005[20]). Environmentally speaking, biomass generated in forest industrial processes, such as sawdust and crusts, is highly polluting. In Mexico, the sawmill industry generates around 0.35 m^3^ of pine sawdust per cubic meter of processed wood (SEMARNAP, 2000[27]). This is equivalent to an annual average sawdust production of 206 thousand cubic meters (INEGI, 2008[8]). Considering an average density of 500 kg/m^3^, such annual production is equivalent to 103 thousand tons (Valencia and López, 1999[33]). On the one hand, the above mentioned by-products reduce the available space in wood transformation centers and therefore, productivity. By-products can also be the cause of an accumulation of dust in the air, with the consequent health risks to sawmill workers and inhabitants of the nearby zones, since dust may bring on a number of respiratory diseases, such asthma, chronic bronchitis, and allergies (Malmström et al., 1999[12]). In addition, dust might be the cause for dermatitis and different kinds of cancer, namely pulmonary, gastrointestinal and nasal (Seguros de Texas, 2004[26]). Moreover, dust has been claimed responsible for other environmental problems, such as fires and spontaneous ignition.

On the other hand, both sawdust and other waste products originated while working with coniferous woods may be raw material for the production of ethanol fuel and other chemical products (Palonen et al., 2004[16]). That is to say that the use of the previously mentioned 103 thousand tons of sawdust would lead to an approximate production of 33x10^6^ L of ethanol fuel. However, the main restriction for producing ethanol fuel from sawdust lies in recovering the sugars that the latter contains. Thus, the recovery stage is determined by the hydrolysis procedure being used (acid or enzymatic). The most investigated pretreatment processes for woody biomass include dilute acid, steam explosion, organosolv, and sulfite pretreatment to overcome recalcitrance of lignocellulose (SPORL) (Zhu et al., 2009[36]). Sodium hydroxide treatment breaks the lignin-carbohydrate bonds, partially removes lignin and hemicelluloses, opens the material structure, increases interface area (Tuor et al., 1995[32]). Ethanol organosolv pretreatment can effectively remove there calcitrance of woody biomass for enzymatic cellulose saccharification (Pan et al., 2005[18], 2006[19]; Pan, 2008[17]). Dilute acid pretreatment is able to mainly hydrolyze hemicelluloses, which turn into a porous material formed by cellulose and lignin (Wyman et al., 2005[35]). Similarly, steam explosion breaks down the structure of the material in order to enhance enzyme access to cellulose (Gregg and Saddler, 1996[7]; Shimizu et al., 1998[28]). The sulfite pretreatment to overcome recalcitrance of lignocellulose (SPORL) is an acidic pretreatment and is very similar to diluted acid pretreatment, in terms of process flow configuration, but with the addition of sulfite or bisulfite. This pretreatment, breaks down the structure of the material and mainly hydrolyze hemicelluloses (Tian et al., 2011[31]). While the HNO_3_ process hydrolyzes and removes lignin from the structure (Wyman et al., 2005[35]), a HNO_3_ and NaOH combination treatments remove a great deal of lignin and hemicellulose and, in a smaller proportion, cellulose; hence a remnant of cellulose material is obtained.

This study reports the results of the evaluation of different pretreatment methods, seeking for the one that leads to higher yields and that enhances both pretreatment conditions and enzymatic hydrolysis to obtain glucose from pine sawdust. The analysis also demonstrates that it is possible to use pine sawdust, considered as a potentially risky pollutant, for the fermentable sugars production, which in turn might be used for producing ethanol fuel.

## Material and Methods

### Lignocellulosic material and chemical composition 

The sawdust used in this study came from *Pinus pseudostrobus* trees that had just been felled under authorization for forest harvesting in the Indigenous Community of San Juan Nuevo Parangaricutiro, in Michoacán, Mexico (19°21´00´´ N. Lat., 102°08´15´´ W. Long.), at 1674 mamsl. The sawdust samples were milled and sieved using a 20-fraction mesh (841 µm) retained on 40 mesh screen. 

The following chemical features were determined in duplicate in this research work: moisture percentage (TAPPI, 2000[30]), pH (Sandermann and Rothkamm, 1959[25]), and ashes (TAPPI, 2000[30]). The total amount of extractives was determined using a Soxhlet extraction sequence with cyclohexane, acetone, methanol, and water. Whereas Runkel lignin (Runkel and Wilke, 1951[21]), holocellulose (Wise et al., 1946[34]), and alpha cellulose (ASTM, 2000[2]) were all determined in extractive-free wood meal.

### Pretreatments for hydrolysis

Sawdust samples were treated according to five pretreatment methods at the specified operating conditions shown in Table 1(Tab. 1). Samples of pure cellulose and pine sawdust, without any pretreatment at all, were used as controls. Most articles are likely to have only two levels of headings. All experiments were performed in duplicate.

#### Alkaline pretreatment

The procedure of González et al. (2011[6]) was performed in this step using a 3 % NaOH concentration, instead of one of 0.5 % NaOH. 10 g samples of sawdust were mixed with 100 mL of sodium hydroxide, ACS grade, in 250-mL-Erlenmeyer flasks. The mixture was then heated in a sterilizer at 121 °C for 78 minutes. The mixture was next set aside to cool to room temperature and after that filtered through a nylon filter cloth. Finally, the solid fractions were squeezed and dried out.

#### Acid pretreatment

10 g sawdust samples were mixed with 100 mL of 5 % H_2_SO_4_ in 250-mL-Erlenmeyer flasks. After that the mixture was heated in a sterilizer at 121 °C for 60 minutes, set aside to cool to room temperature, and filtered. The solid fractions were squeezed, dried out and weighed (Cortez, 2010[5]).

#### Steam explosion

10 g sawdust samples were mixed with 100 mL of distilled water in 250-mL-Erlenmeyer flasks, which were capped and left to stand for 48 hours at room temperature. Next, the samples were heated in a sterilizer (at 121 °C), and a sudden decompression of the system was made. Flasks were allowed to cool to room temperature. The solid fraction was filtered through a nylon cloth, dried out, and weighed (Oliva, 2003[15]). 

#### Organosolv pretreatment

This pretreatment was performed using a reflux equipment in which the 10 g sawdust samples was treated with watery ethanol and acetone (60/40 w/w), with a solid-liquid ratio of 7:1, at 80 °C for 90 minutes. At the end of the treatment the samples were allowed to cool to room temperature and filtered through nylon cloth; the solid fraction was then squeezed, dried out, and weighed (Martinez, 2011[13]).

### Combined nitric acid and sodium hydroxide

20 g sawdust samples were mixed with 120 mL of 6 % nitric acid in a ball flask. The mix was kept under reflux conditions at 100 °C for 60 minutes in an oil bath. The solid fraction was washed out with distilled water, allowed to dry, and later mixed with 120 mL of 1 % sodium hydroxide. The mixture was heated at 90 °C for 30 minutes under reflux conditions (Alhasan et al. 2010[1]).

### Enzymatic hydrolysis

Pretreated materials were hydrolyzed using a commercial enzymatic complex obtained from *Trichoderma reesei *(Celluclast 1.5 L, a product of NOVOZYME Corp.). For the hydrolysis step, 1 g samples of pretreated material were mixed with the appropriate amount of enzymatic extract in 20-mL-containers to provide 25 FPU. Meanwhile, the mix pH was adjusted to 4.5 by adding the required volume of 0.1 molar buffer acetate solutions until reaching 10 mL. The mixture was later shaken for 72 hours, at 80 rpm and 45 °C. The following step was taking samples of the hydrolyzed material every twelve hours, determining their reducing sugar content using Chaplin and Kennedy´s DNS method (Chaplin and Kennedy, 1994[4]).

The yield results were used to evaluate the pretreatments procedures. A variance analysis was conducted with a 95 % confidence level. Both the LSD method and the multiple rank tests were applied. Data were processed with STATGRAPHICS Centurion XVI software, version 16.1.18.

### Identification of the hydrolysis variables

Out of the evaluated pretreatments, the one with the greatest yield was the combined pretreatment. After applying a 2^3^ factorial design, the factors and levels obtained were as following: 

factor A: time (30 and 60 min); 

factor B: nitric acid concentration (3 and 6 %); and, 

factor C: temperature (50 and 100 °C). 

The dependent variable was the yield of fermentable sugars. The collected data were used for an analysis of variance (ANOVA), with a 95 % confidence level. The software used was STATGRAPHICS Centurion XVI, version 16.1.18.

### Maximizing reducing sugar yield

The results from the 2^3^ factorial design showed that the pretreatment that yielded to the highest concentration of reducing sugar (36.55 g/L) was the one performed with 6 % nitric acid at 100 °C for 30 minutes. Hence, to maximize the hydrolysis yield a 3^2 ^factorial design was used and all the experiments were run in duplicate. The factors and levels were: 

factor A: Nitric acid concentration (4.5, 6 and 7.5 %); and, 

factor B: temperature (80, 100 and 120 °C); pretreatment time was kept constant at 30 minutes. 

Collected data were used for an analysis of variance (ANOVA), with a 95 % confidence level. The software used was STATGRAPHICS Centurion XVI, version 16.1.18.

### Calculations

The yield obtained for each pretreatments procedure was expressed in terms of weight by means of Eq. 1,

***RP = [(W_i_-W_f_) / W_i _]*** **x 100** [1]

where: *W_i _*(g) is the weight of lignocellulosic material subjected to pretreatment, *W_f_* (g) is the weight of pretreated material, and *RP* is the pretreatment yield (% on a mass basis).

The production of reducing sugars obtained by means of enzymatic hydrolysis of pretreated materials was quantified by Eq. 2.

**% *RA* = *[(A x W_1_ x 0,9) / B ]* x 100** [2]

where: *A *(g) is the amount of reducing sugars in the hydrolyzed material, *W_1 _*(g) is the initial weight of the sample that underwent hydrolysis, B (g) is the amount of cellulose on untreated wood, and *RA *is the cellulose hydrolysis yield (%).

## Results and Discussion

### Chemical composition 

Table 2(Tab. 2) shows the results of the chemical composition of pine sawdust. The observed results match those data reported by other authors for the same wood species (Rutiaga-Quiñones, 2001[22]; Lima-Rojas, 2013[9]).

### Pretreatments for hydrolysis

Table 3(Tab. 3) shows different yields obtained in each pretreatment tested. The reducing sugars yield in the liquor produced during the acid pretratment was low (18.2 % or 18.2 g/L). The results displayed in Table 3(Tab. 3) on column “Reducing sugar yield (%)” are based on the 6 carbon carbohydrates on untreated wood (Equation 2) and clearly show that the pretreatment with the highest hydrolysis yield of reducing sugars was the combined pretreatment, performed with 6 % nitric acid at 100 °C, for 60 minutes. There was some considerable weight loss of material (59.16 %), though.

Data collected for the alkaline pretreatment in this work were similar to those obtained by Saha and Cotta (2006[23]) for pretreated wheat straw (8.6 % w/v) with alkaline peroxide (2.15 % of H_2_O_2_ v/v, pH 11.5, 35 °C, 24 h) and for an enzymatic saccharification (45 ºC, pH 5.0, 120 h) with three commercial enzyme preparations (cellulase, beta-glucosidase, and xylanase).

Sugar yields obtained by hydrolysis with sulfuric acid were lower than those reported by Saha et al. (2005[24]), who used rice husk pretreated with sulfuric acid (7.83 % w/v, 0.75 % of H_2_SO_4_ v/v, 60 min, 121°C) prior to hydrolysis with a mixture of cellulase and glucosidase (Celluclast, Novozyme 188), for 72 h at 121 °C. However, the results obtained in this work were higher to the ones reported by Awasthi et al. (2013[3]), who treated water lilies with sulfuric acid (4 % v/v, 121 °C, 15 minutes) and reported 12.63 mg of reducing sugars per gram of water (carbohydrates in watery solution).

Pretreatment using steam explosion led to lower yields than those obtained by López-Miranda et al*.* (2009[10]), who reported an 8.15 % of reducing sugars after pretreating sawdust pine with steam explosion for 30 minutes 120 °C, at hydrolysis pH of 4.5, for 240 h, with an enzymatic load of 25 FPU/g of total carbohydrates and lower than Shuai et al. (2010[29]) who reported 77.7 % at 180 °C for 30 min using steam explosion and sulfuric acid loading of 5 % on oven-dry wood and a 5:1 liquor to-wood ratio whit spruce wood and enzyme loading of 15 FPU.

Regarding organosolv pretreatment, Mesa et al. (2011[14]) reported higher yield than those obtained in this study. They treated sugarcane bagasse (29.1 % glucose) with 30 % (v/v) ethanol at 195 °C, for 60 minutes. The difference is due to: 1) this process was previously pretreated with diluted acid; 2) there was a later addition of NaOH; and 3) the difference in temperature at which the process was carried out.

Finally, the yields obtained with combined pretreatment were a little lower than those obtained by Alhasan et al. (2010[1]) with rubber tree wood pretreated with 6 % HNO_3_ at 100 °C for 60 minutes, followed by 1 % NaOH plus cellulase along with β-glucosidase with 25 FPU and 60 UBC, respectively. Our sugar yields obtained with combined pretreatment was also a little lower than those obtained by Luo et al. (2010[11]), who used the SPORL treatment with the following experimental conditions: a chemical charges sulfuric acid (2.21 %) and sodium bisulfite (8 %) on oven dry pine wood, enzimatic charge of Celluclast 1.5 L (15 FPU/g substrate) and Novozyme 188 (22.5 CBU/g substrate). 

On the other hand, sugars yield at the specified conditions using the sodium hydroxide pretreatment was 24.76 % higher than the sawdust control sample without pretreatment. The sulfuric acid pretreatment led to the second lowest reducing sugars results after enzymatic hydrolysis (Table 3(Tab. 3)). Nevertheless, while performing this pretreatment, the amount of reducing sugars in the aqueous solution was 18.2 %, which was 87.05 % greater than the yield of the sawdust control sample in the solution at the start of the pretreatment. As can be seen from Table 3(Tab. 3), organosolv and steam explosion pretreatments, as well as the sawdust sample without pretreating (control sample) exhibited very close results in their corresponding yields of reducing sugars. Therefore, steam explosion and organosolv were not effective pretreatment methods for pine sawdust prior to an enzymatic hydrolysis. The combined pretreatment led to the highest production of reducing sugars after hydrolysis (Table 3(Tab. 3)), 341.5 % greater than the sawdust sample pattern and 47.8 % greater than the cellulose control sample.

When comparing the saccharification yield obtained with the combined pretreatment and the other methods used in this study, it was found that such production was higher in all the cases. With sodium hydroxide it was 273.7 % higher, with sulfuric acid pretreatment is was 182.6 % higher, and with steam explosion it was 385.5 % higher. 

This seems to suggest that a consecutive pretreatment with nitric acid and sodium hydroxide facilitates enzymatic hydrolysis of the pretreated material, since nitric acid helps to partially hydrolyze lignin and hemicelluloses, and sodium hydroxide favors the release of lignin and an increasing amount of amorphous cellulose content in the material. All of the above contribute to a higher reducing sugar concentration in the final product. 

### Identification of hydrolysis variables

It follows from the factorial design 2^3^ results that factor A, time, did not have any significant statistical effect over sugars yield (p=0.1345), whereas factors B and C, concentration of nitric acid and temperature, did have an effect (p=0.0000). These results show that the highest sugars yield (91.26 %) was obtained using the 6 % nitric acid pretreatment, at 100 °C for 30 minutes (7^th^ run) (Table 4(Tab. 4)). As observed, acid concentration and operating temperature were the same as those reported in Table 1(Tab. 1) (before actually beginning the experiments), but in this case time was reduced to 30 minutes, which means a 50 % saving in processing time. Hence, an operating cost reduction opportunity can be spotted when processing this raw material. 

### Maximization 3^k^

The factorial design 3^2^ results indicate that the maximum reducing sugars yield was obtained pretreating sawdust with 7.5 % nitric acid, at 120 °C for 30 minutes (Table 5(Tab. 5)). Such yield is even higher than that obtained with the pure cellulose control sample (Table 1(Tab. 1)). 

## Conclusions

After comparing the pretreatment methods evaluated in this study, the combined method (with nitric acid and sodium hydroxide) yielded to the best results and it went up to the maximization of getting to pretreatment, the second finding was that the maximum reducing sugars yield (97.83 ± 1.59 %) from raw material was obtained when performing the 7.5 % nitric acid pretreatment, at 120 °C, followed by the 1 % sodium hydroxide at 90 °C, according to the 3^2 ^factorial design applied. 

## Acknowledgements

The authors are grateful both to CIC-UMSNH (Project 21.3-JGRQ) and CONACYT (Project 16644) for all support provided, and deeply appreciate the study material donated by the Indigenous Community of San Juan Parangaricutiro, Michoacán. 

## Declaration of interest

The authors declare that they have no conflict of interest. The authors are alone responsible for the content and writing of the paper. 

## Figures and Tables

**Table 1:**
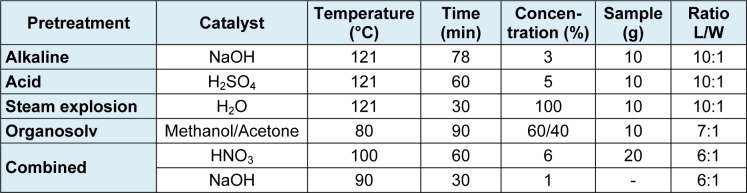
Pretreatments and specified conditions

**Table 2:**
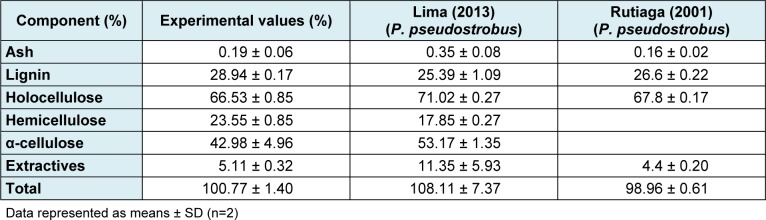
Chemical composition of pine sawdust (w/w)

**Table 3:**
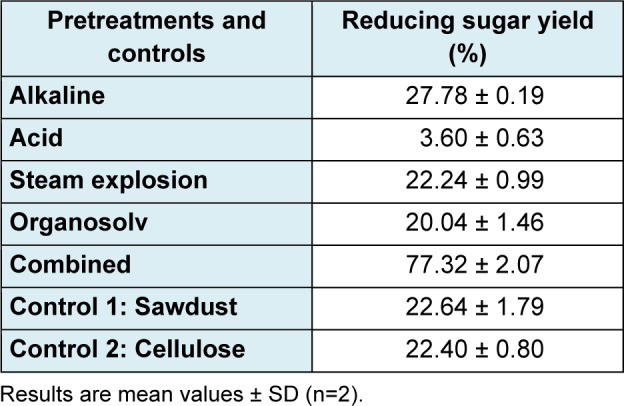
Sugar yields in studied pretreatments and controls (w/v)

**Table 4:**
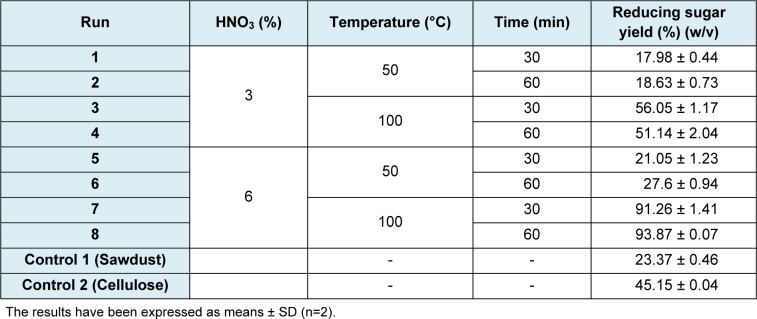
Design matrix (2^3^) of hydrolysis and results

**Table 5:**
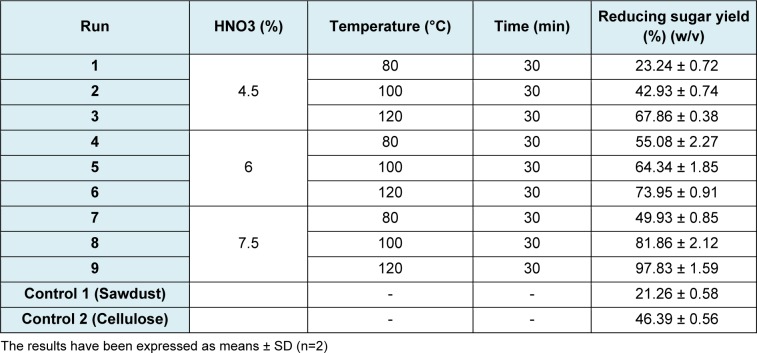
Design matrix (3^2^) of hydrolysis and results
